# Supplementary material for: P-TRAP: a Panicle Trait Phenotyping tool
Source: BMC Plant Biol. 2013 Aug 29;13:122. doi: 10.1186/1471-2229-13-122 (PMC3848748; doi:10.1186/1471-2229-13-122)
Supplement: Additional file 7 — P-TRAP source code. The Netbeans project that contains the source code of the application. [file 1471-2229-13-122-S7.zip › javadoc.zip!/javadoc/org-basmati-workspace/org/basmati/workspace/ImageViewScene.html]

ImageViewScene (WorkSpace)


JavaScript is disabled on your browser.


- Package
- Class
- Tree
- Deprecated
- Index
- Help

*org.basmati.workspace 1.0*

- Prev Class
- Next Class

- Frames
- No Frames

- All Classes

- Summary:
- Nested |
- Field |
- Constr |
- Method

- Detail:
- Field |
- Constr |
- Method


org.basmati.workspace

## Class ImageViewScene

- java.lang.Object
- - org.netbeans.api.visual.widget.Widget
  - - org.netbeans.api.visual.widget.Scene
    - - org.basmati.workspace.ImageViewScene

- All Implemented Interfaces:
  :   javax.accessibility.Accessible, org.openide.util.Lookup.Provider

  ---

    

  ```
  public class ImageViewScene
  extends org.netbeans.api.visual.widget.Scene
  ```

  The Scene that shows the input images. It allows the user to do some basic operation like cropping and selecting the scale.

  Author:
  :   faroq

- - ### Nested Class Summary

    - ### Nested classes/interfaces inherited from class org.netbeans.api.visual.widget.Scene

      `org.netbeans.api.visual.widget.Scene.SceneListener`
    - ### Nested classes/interfaces inherited from class org.netbeans.api.visual.widget.Widget

      `org.netbeans.api.visual.widget.Widget.Dependency`
  - ### Constructor Summary

    Constructors

    Constructor and Description

    `ImageViewScene()`
  - ### Method Summary

    Methods

    | Modifier and Type | Method and Description |
    | --- | --- |
    | `void` | `addImage(java.awt.Image image)` |
    | `void` | `createLabel()` |
    | `void` | `createSelectionSquare(java.awt.Rectangle rectangle, int handelsize)` Create the selection frame |
    | `java.awt.Rectangle` | `getSelectionBounds(int handelsize)` |
    | `void` | `selectScale(long pixels)` Set the scale on the input image. |
    | `void` | `setupActions()` |
    | `void` | `setuplayers()` |

    - ### Methods inherited from class org.netbeans.api.visual.widget.Scene

      `addSceneListener, convertSceneToView, convertSceneToView, convertViewToScene, createBirdView, createSatelliteView, createView, createWidgetHoverAction, getActiveTool, getDefaultFont, getFocusedWidget, getGraphics, getInputBindings, getKeyEventProcessingType, getLookFeel, getMaximumBounds, getPriorActions, getResourceTable, getSceneAnimator, getView, getZoomFactor, isRepaintRequiredForRevalidating, isValidated, paint, removeSceneListener, setActiveTool, setFocusedWidget, setKeyEventProcessingType, setLookFeel, setMaximumBounds, setResourceTable, setZoomFactor, validate, validate`
    - ### Methods inherited from class org.netbeans.api.visual.widget.Widget

      `addChild, addChild, addChild, addChild, addChildren, addDependency, bringToBack, bringToFront, calculateClientArea, convertLocalToScene, convertLocalToScene, convertSceneToLocal, convertSceneToLocal, createActions, equals, getAccessibleContext, getActions, getActions, getBackground, getBorder, getBounds, getChildConstraint, getChildren, getClientArea, getCursor, getCursorAt, getDependencies, getFont, getForeground, getLayout, getLocation, getLookup, getMaximumSize, getMinimumSize, getParentWidget, getPreferredBounds, getPreferredLocation, getPreferredSize, getScene, getState, getToolTipText, hashCode, isCheckClipping, isEnabled, isHitAt, isOpaque, isPreferredBoundsSet, isVisible, notifyAdded, notifyBackgroundChanged, notifyFontChanged, notifyForegroundChanged, notifyRemoved, notifyStateChanged, paint, paintBackground, paintBorder, paintChildren, paintWidget, removeChild, removeChildren, removeChildren, removeDependency, removeFromParent, repaint, resolveBounds, revalidate, revalidate, setAccessibleContext, setBackground, setBackgroundFromResource, setBorder, setBorder, setCheckClipping, setChildConstraint, setCursor, setEnabled, setFont, setFontFromResource, setForeground, setForegroundFromResource, setLayout, setMaximumSize, setMinimumSize, setOpaque, setPreferredBounds, setPreferredLocation, setPreferredSize, setState, setToolTipText, setVisible, updateResources`
    - ### Methods inherited from class java.lang.Object

      `clone, finalize, getClass, notify, notifyAll, toString, wait, wait, wait`

- - ### Constructor Detail


    - #### ImageViewScene

      ```
      public ImageViewScene()
      ```
  - ### Method Detail


    - #### setuplayers

      ```
      public void setuplayers()
      ```


    - #### setupActions

      ```
      public void setupActions()
      ```


    - #### addImage

      ```
      public void addImage(java.awt.Image image)
      ```


    - #### createSelectionSquare

      ```
      public void createSelectionSquare(java.awt.Rectangle rectangle,
                               int handelsize)
      ```

      Create the selection frame

      Parameters:
      :   `rectangle` - A `Rectangle`
      :   `handelsize` - the size of the frame size.


    - #### getSelectionBounds

      ```
      public java.awt.Rectangle getSelectionBounds(int handelsize)
      ```


    - #### createLabel

      ```
      public void createLabel()
      ```


    - #### selectScale

      ```
      public void selectScale(long pixels)
      ```

      Set the scale on the input image.

      Parameters:
      :   `pixels` -


- Package
- Class
- Tree
- Deprecated
- Index
- Help

*org.basmati.workspace 1.0*

- Prev Class
- Next Class

- Frames
- No Frames

- All Classes

- Summary:
- Nested |
- Field |
- Constr |
- Method

- Detail:
- Field |
- Constr |
- Method
